# Nasal Irrigations: A 360-Degree View in Clinical Practice

**DOI:** 10.3390/medicina61081402

**Published:** 2025-08-01

**Authors:** Luca Pecoraro, Elisabetta Di Muri, Gianluca Lezzi, Silvia Picciolo, Marta De Musso, Michele Piazza, Mariangela Bosoni, Flavia Indrio

**Affiliations:** 1 Pediatric Unit, Ospedale Vito Fazzi, ASL Lecce, 73100 Lecce, Italy; 2 Pediatric Department, University of Bari Aldo Moro, 70121 Bari, Italy; 3 Pediatric Unit, Department of Surgical Sciences, Dentistry, Gynecology and Pediatrics, University of Verona, 37129 Verona, Italy; 4 Private Practice Pediatric Allergist, 20100 Milan, Italy; 5 Department of Experimental Medicine Pediatric Section, University of Salento Hospital “Vito Fazzi”, 73100 Lecce, Italy

**Keywords:** nasal irrigation, upper respiratory tract infections, mechanical cleansing, viral load reduction, sinonasal disorders

## Abstract

Nasal irrigation (NI) is an effective, safe, low-cost strategy for treating and preventing upper respiratory tract diseases. High-volume, low-pressure saline irrigations are the most efficient method for removing infectious agents, allergens, and inflammatory mediators. This article reviews clinical evidence supporting NI use in various conditions: nasal congestion in infants, recurrent respiratory infections, acute and chronic rhinosinusitis, allergic and gestational rhinitis, empty nose syndrome, and post-endoscopic sinus surgery care. NI improves symptoms, reduces recurrence, enhances the efficacy of topical drugs, and decreases the need for antibiotics and decongestants. During the COVID-19 pandemic, NI has also been explored as a complementary measure to reduce viral load. Due to the safe profile and mechanical cleansing action on inflammatory mucus, nasal irrigations represent a valuable adjunctive treatment across a wide range of sinonasal conditions.

## 1. Introduction

**Figure 1:**
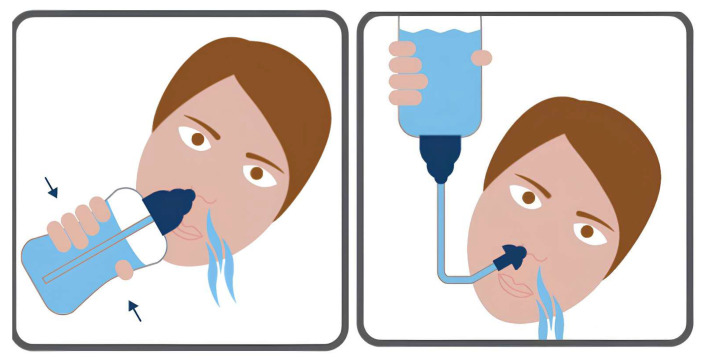
Optimal head position for effective NI.

Nasal irrigation (NI) is an ancient healing practice of the upper respiratory tract that originated in the medical tradition from the ancient Hindu practice of Ayurveda, whose roots go back to the Vedas, a thousand years before Christ. Historically, “Neti” is an integral part of the yogic system of body cleansing techniques [1]. It is primarily intended to cleanse the first airways, the nose, throat, and pharynx, and has been used for thousands of years to relieve rhinosinusitis and allergy symptoms. The two main variants of Neti are those which use water poured into a nostril so that it comes out of the nostril against the side, and the more advanced sutra neti. “Sutra Neti” represents an alternative NI. It consists of a piece of wet string inserted through the nose into the mouth; holding both ends simultaneously, the string is alternately pulled in and out of the nose and pulled out of the mouth at the end of the procedure. Both techniques are used to rinse the nasal cavities, using gravity to make the water flow through the nose [2]. NI was introduced into Western medicine at the beginning of the 19th century [3] and has continued to gain popularity worldwide [4]. It is used alone or with other therapies in various conditions, including acute and chronic rhinosinusitis [5,6,7,8] and allergic rhinitis [9,10]. Furthermore, especially in children, it is prescribed to treat and prevent upper respiratory tract infections [11]. In general, otorhinolaryngologists and paediatricians play a crucial role in adopting NI as they consider it very effective. It is associated with significantly reducing the signs and symptoms of rhinosinus pathologies and prescribing drugs commonly used in these conditions [11,12]. Their endorsement and recommendation of NI can dramatically influence its acceptance and use in clinical practice. NI’s definition and application remain imprecise, especially in the paediatrics population. The most common modalities used include drops, sprays, and syringes. The most widely used devices are syringes, but they have some limitations, because part of the solution may leak from the nostril before reaching the nasal cavity. In addition, pressure can be different depending on the power applied by the operator [12]. The most used solutions are isotonic saline (0.9%) and hypertonic saline (1.5–3%), both with an acidic pH (4.5–7). Concentrations above 3% are not recommended due to the dose-dependent complications such as pain, congestion, and rhinorrhoea [13]. A recent study showed that adding xylitol to the solution is not recommended as first-line treatment for paediatric chronic sinusitis (CRS), while low-volume, low-pressure hypertonic irrigation, twice a day for 6 weeks, has been shown to be safe and effective [14]. Some studies suggest that there are preferred positions for performing nasal irrigation manoeuvres: for children, an effective technique is the “fencing” method, which involves supine positioning with a 30° tilt and the head turned sideways to prevent aspiration or regurgitation [15] (Figure 1).

## 2. The Upper Respiratory Tract: Barrier and Cleansing Mechanisms

### 2.1. The Upper Respiratory Tract Is the Gateway for Pathogens

The upper respiratory tract is the gateway for viruses and bacteria to infect the respiratory system. The nose and throat are their first site of impact, adhesion, and infection. In a study conducted in the state of Virginia in the USA and carried out at nine different locations, it was calculated that the concentrations of both virus- and bacteria-like particles are approximately 10^5^ particles per m^3^ of air, and that outdoor concentrations are 2.6 and 1.6 times higher than those indoors, respectively [16]. While resting, a normal adult inhales about 8 litres of air per minute, roughly equivalent to 11,520 litres—11.52 cubic metres of air—per day. So, a man who spends the whole day resting at home inhales 51,000 bacteria/cubic metre × 11.52 cubic metres/day = 590,000 bacteria/day [17]. In addition to microorganisms, atmospheric pollutants, particularly ultra-fine dust, can negatively affect the respiratory system. These dust particles are absorbed in the lungs and distributed through the circulatory system to the entire organism, with harmful effects on the cardiovascular system, especially in individuals with little defence against oxidative stress [18]. The respiratory tract is structured to defend itself with anatomical and functional features that help it to rid itself of pollutants and potential pathogens. A lot of evidence is known. First, the nasal cavity has a mucus ciliary lining. Second, the inside of the nose is lined with hair (absent in young children) that filters out particles larger than 5–10 µm in diameter. Third, the nasal turbinates are covered with mucus that retains particles not filtered by the nasal hairs. Fourth, the change in the direction of airflow from the sinuses to the pharynx causes many larger particles to hit the back of the throat and be swallowed. Fifth, adenoids and tonsils function as mechanical and immunological barriers. The development of an infection requires several predisposing conditions. First, enough infectious agents must be present in the air and inhaled. Second, the pathogens must remain alive and viable after entering the respiratory tract. Third, viruses and bacteria need to reach the host’s susceptible tissues, causing their colonization. The loss of the protective function of the nasal mucosa contributes to the onset of upper respiratory tract infections. This represents the biological rationale for nasal irrigation, which serves to reduce the concentration of airborne pollutants and microorganisms, consequently compromising their viability and ability to colonize the host. Under normal physiological conditions, the epithelial cells of airways are linked by junctional complexes that form an effective barrier against pathogens. These cells also secrete mucus, which is propelled by the coordinated movement of cilia toward the glottis, where it is swallowed and neutralized at the gastric level. Moreover, the respiratory mucosa is impregnated with defence peptides. It expresses recognition receptors of the innate immune system to respond rapidly and non-specifically to any foreign substance and all inhaled pathogens. All these defence mechanisms are compromised by air pollutants, which increase the risk of infection and exaggerated inflammatory responses. The mechanical and immunological changes induced by air pollution are determined by the activation of free radical-sensitive pathways, with the consequent impairment of antioxidants balance [19].

### 2.2. The Upper Respiratory Tract as the Gateway for Allergens

The prevalence of pollen allergy in the European population is up to 40%. It is the most common allergen in Europe [20]. Even low pollen concentrations in the air can cause allergic symptoms in very sensitive people. The purification of the first grass pollen allergen in 1965 by David Marsh [21] enabled the first specific investigation of the properties of pollen grains relevant to allergy development. Furthermore, Marsh provided accurate estimates of inhaled allergen amounts (approximately 10 ng/day, equivalent to about 1 mcg/ in a pollen season) and demonstrated that pollen allergens elute rapidly from the granules in aqueous solution and when deposited on our mucous membranes [22]. This emphasizes the role of nasal irrigation in managing allergies, as it effectively reduces the concentration of allergens, thereby relieving those sensitive to them. The European Academy of Allergy and Clinical Immunology (EAACI) fixes the beginning of the season for various pollen species based on their air concentration and their effects on human health. The beginning of the grass pollen season, for example, is defined when 5 out of 7 consecutive days carry more than 10 graminaceous pollen grains/m^3^ of air, and the sum of pollens on these 5 days is more than 100 graminaceous grains/m^3^ of air [23]. Emergency room visits and hospital admissions increase when grass pollen concentrations exceed 10–12 grains/m^3^ of air [24]. Similar criteria exist for cypress, birch, olive, and ragweed [25] When an adult is outside, he/she inhales approximately 50 to 100 pollen grains per day. The most important allergens in the home environment are the faecal particles of Dermatophagoides pteronyssinus and Dermatophagoides farinae. These mites feed mainly on human and animal dander and are particularly at home in warm places with a relative air humidity of over 50%. This is why they mostly live in mattresses, blankets, pillows, upholstered furniture, and carpets, where they deposit their allergens. Each faecal particle is similar in size to pollen grains, has a diameter of 20–40 μm, and contains about 0.2 ng of major allergens. Mite faecal particles, relative to their size, do not remain airborne. Therefore, we inhale them—5 to 100 per night—when we disturb them—in practice, when we move around in bed or clean carpets [26]. The amount of inhaled mite allergen is generally lower (≤10 ng/day) than that of pollen [27]. Still, its concentration in faecal particles can be very high, about 0.2 ng in a 20-micron sphere, roughly equivalent to 2 mg/mL. The mite’s major allergen, Der p 1, in aqueous solution, as on respiratory mucous membranes, elutes rapidly from the particles: 90% in 2 min [28]. Therefore, its effects can be very significant at the impact site of the particle, where it induces localized inflammation and a concomitant increase in bronchial reactivity [26]. Generally, patients with mite allergies do not complain of symptoms when they are in bed. When a second stimulus, particularly a viral airway infection, occurs, it involves all of the bronchial tree [29,30]. Consequently, the patient could present a bronchospasm even in a non-asthmatic state [31]. In this case, environmental prophylaxis with mite covers is fundamental [32]. Exposure to cat allergens dramatically differs from mite or pollen allergens because Fel d 1, the primary cat allergen, remains dispersed most of the time in the air of homes with a cat [33]. It has been estimated that a child can inhale up to 1 mcg Fel d 1⁄ day, i.e., as much as 100 times the amount of mite or pollen allergens [34]. Cat allergens are distributed throughout the bronchial tree, and the asthma patient immediately experiences dyspnoea. The inflammation of the nasal mucosa, caused by allergens and microorganisms, is aggravated by exposure to atmospheric pollutants that, like viruses and bacteria, we can hardly avoid inhaling. The aim is to reduce the additive effect of these noxae by reducing their concentration in the nasal cavities.

### 2.3. The Mechanism of Action of NI

The exact mechanism of action of NI is not entirely known. It is known that nasal lavage can result in an improvement in nasal mucosal function through various physiological effects: First, the removal of sticky secretions [35,36,37]. Second, the dilution and removal of mediators of inflammation, such as histamine and prostaglandins, which can become trapped in mucus [38,39]. Third, the restoration and improvement of mucociliary function and increased effectiveness of ciliary beating [40,41]. Fourth, the prevention of secondary infections and promoting mucosal healing [42,43]. Fifth, the improvement of barrier effect against bacteria and viruses [44,45,46]. In addition, a topical antibacterial action of hypertonic saline solution has been documented [46,47,48,49]. Starting from this evidence, NI is also believed to play an essential role in the postoperative period of chronic sinusitis refractory to medical therapy because it reduces the risk of adhesions and promotes stomatal patency [41]. This latter effect is beneficial because chronic sinusitis is associated with a worsening of ciliary clearance due to both osmotic changes in the mucus layer and a reduction in the frequency of the ciliary beat itself [41,42]. The possibility of administering drugs by nasal nebulization has also been suggested: direct contact with the mucosa favours higher local concentrations and fewer systemic effects than oral administration [50].

## 3. Demonstrated Use of NI in Clinical Practice

To effectively eliminate microorganisms, pollutants, and chemical mediators of inflammation, the mucous membranes of the nose need to be “washed” with a sufficient volume of water; this may seem rudimentary, but it is highly effective. Therefore, the optimal method for conducting nasal irrigation (NI) employs a low-pressure, high-nasal shower [51,52]. This method is regarded as the “gold standard” for nasal irrigation due to its ease of use, affordability, availability, tolerability, and superior distribution of saline solution within the sinuses [53,54]. Low-volume devices, such as sprays and pre-packaged squeeze bottles, are generally less effective and more costly; they tend to moisten the nose without providing an adequate “washing” action [55]. Additionally, the nasal shower enables users to prepare customized solutions and can serve as a drug delivery system for medications like antibiotics and high-dose topical steroids [51]. Numerous clinical conditions have demonstrated the efficacy of NI, whether as a standalone treatment, an adjunctive therapy, or a preventive strategy.

### 3.1. Infants with Nasal Congestion

A recent Delphi consensus, a structured expert consensus process, endorsed nasal irrigation (NI) for treating infant nasal congestion [52,56]. Infants with bronchiolitis benefit significantly: nasal obstruction increases respiratory effort, which NI [57] and suctioning [58] can mitigate. NI also improves sleep quality and feeding [56]. Experts recommend NI whenever difficult nasal breathing is observed, with no consensus on optimal frequency, though 66.7% favoured once-daily use for infants with comorbidities (cardiac, respiratory, or neurological) [52]. Prophylactic NI in healthy infants remains debated.

### 3.2. Prevention of Respiratory Infections

Upper respiratory tract infections (URTIs) and sinus symptoms represent common health issues, particularly in children [59], with sinusitis prevalence reaching 32% in this population [60]. This high incidence frequently leads to antibiotic prescriptions [61]. NI with saline solution emerges as a cost-effective intervention that reduces medication use and associated costs while potentially decreasing antibiotic resistance [62]. A survey of nearly 1000 Italian paediatricians revealed that 75% consider NI effective and well-tolerated for prevention and treatment [10,63]. In a study of 400 children (aged 6–10 years) with uncomplicated colds/flu, NI combined with standard therapy showed a faster resolution of acute nasal symptoms, reduced recurrence rates, lower symptom scores (sore throat, cough, obstruction, nasal discharge), fewer sick days (31% vs. 75% in controls), reduced school absences (17% vs. 35%), and lower complication rates (8% vs. 32%) [11]. Moreover, a cross-sectional study involving 2386 schoolchildren and 519 preschoolers demonstrated that NI reduced acute respiratory infection rates by 2.4–3.2 times during epidemics while improving outcomes in URTIs and asthma [64]. This benefit of NI was also demonstrated in adults. Performing NI daily for 20 weeks during the cold season prevented cold symptoms in 100 healthy participants [65]. Another tool for preventing respiratory infections is represented by hypertonic solutions (1.5–3% saline). They provide superior antimicrobial effects by stimulating secretion of antimicrobial neuropeptides (e.g., substance P) [66,67,68], releasing LL-37 cathelicidin from glycosaminoglycan complexes [69], and utilizing salt’s inherent antibacterial properties (historically used in food preservation). For young children, 1.5–2% solutions offer an optimal balance of efficacy and tolerability. Paediatricians often recommend post-school NI to reduce household transmission, complementing hand hygiene measures [70].

### 3.3. Recurrent Acute Respiratory Infections

Children experience an average of 10 respiratory tract infections (RTIs) in their first 3 years of life [71]. Nasal discharge, a common manifestation, persists for approximately 2 months annually in infants [72]. Anatomical factors (narrower airways, higher nasal resistance) make infants particularly susceptible to mucopurulent rhinorrhoea, feeding difficulties (sucking–swallowing impairment) [73], and recurrent otitis media [74]. NI represents a safe, cost-effective intervention for paediatric upper airway management [52,62]. The use of NI in the context of recurrent acute respiratory infections demonstrated a reduction in rhinologic symptoms and a decreased incidence of acute rhinosinusitis and complications [75]. Specifically, a case–control study involving 75 children compared 15 days/month for 3 months, comparing saline vs. saline + hyaluronic acid. The hyaluronic acid group showed significant improvements in ciliary motility (OR = 13.61; 95% CI 4.51–41.00; *p* < 0.001), adenoid hypertrophy resolution (OR = 14.72; 95% CI 4.74–45.68; *p* < 0.001), bacterial clearance (OR = 2.95; 95% CI 1.15–7.55; *p* = 0.026), neutrophil reduction (OR = 4.51; 95% CI 1.75–11.62; *p* = 0.002), rhinitis duration (OR = 10.47; 95% CI 3.10–35.31; *p* = 0.040), nasal obstruction (OR = 3.80; 95% CI 1.09–13.19; *p* = 0.047), and biofilm formation (OR = 9.90; 95% CI 2.61–37.47; *p* = 0.049) [76] The rhinologic symptoms’ improvement was demonstrated also in adults. When NI is administered within 48 h of rhinologic symptom onset, it reduces symptom duration, decreases OTC medication use, lowers viral load, and reduces household transmission [46]. The best application of NI in the context of recurrent respiratory infection seems to be represented by pathogens with prolonged incubation periods and localized upper respiratory infections, conditions where viral load correlates with disease severity [77] In recent years, nose washing and gargling have represented a demonstrated prevention strategy and complementary therapy against SARS-CoV-2 [78,79,80]. It is known that the nasal epithelium is the primary site for viral entry, replication, and shedding [81,82]. Given the rapid viral replication cycle (initial release within 6 h post-infection [83]), routine NI during high-risk exposures (e.g., healthcare settings) may offer prophylactic benefits [78]. This was demonstrated in elderly patients, who performed a twice-daily NI with 250 mL hypertonic solution within 24 h of a positive detection of SARS-CoV-2, reducing the hospitalization risk by eight times. In addition, mortality was 0% vs. 1.5% in controls [84]. A dose–response relationship was demonstrated. Specifically, 80% of twice-daily users reported no/mild symptoms vs. 42% with less frequent use. On the other hand, controls experienced persistent symptoms for 2–3 weeks in more than 50% of cases [85]. Starting from this evidence, it was estimated that there was a decline in hospitalization rates from 11% to 1.3%, preventing ~1 million hospitalizations among elderly Americans [86,87]. This evidence was also demonstrated in healthcare workers who performed regular nasal/oral rinsing: they reduced symptomatic infections to 1.2% vs. 12.7% in controls (*p* = 0.0039) [87]. The physiopathological basis of the benefit of NI in this context is related to the hypertonic NI’s antiviral effects. Many reasons explain it. First, hypochlorous acid (HOCl) generation: nasal mucosal cells convert NaCl’s chlorite ions to HOCl, a potent antiviral agent also used in SARS-CoV-2 disinfectants [46,88]. Second, a direct virucidal action: halide salts inhibit RNA viruses in vitro [88,89], mirroring salt’s historical role as a preservative. Starting from this biological evidence, the clinical practice is based on an early NI post-exposure for maximizing viral load reduction [86]. On the other hand, efficacy and tolerability must be balanced using hypertonic solutions (e.g., 1.5–3%) [84,87].

### 3.4. Acute Sinusitis

Generally, acute rhinosinusitis primarily begins as a viral infection, frequently progressing to bacterial superinfection. This condition involves concurrent inflammation of the nasal and sinus mucosa [90]. While the traditional treatment relied on antibiotics and corticosteroids [91], further evidence demonstrated the role of NI for children and adults [36,92]. Hyaluronic acid-enriched saline solutions are adjuvant because they reduce rhinologic symptoms and illness duration [93]. Specifically, the 2020 EPOS guidelines strongly encourage using saline NI in rhinosinusitis [94], from initial self-care to post-surgical recovery. These recommendations reflect extensive clinical evidence demonstrating NI’s role in preventing recurrent episodes and reducing chronicity risk [95].

### 3.5. Acute Recurrent Sinusitis

The 2015 American Academy of Otolaryngology–Head and Neck Surgery Foundation guidelines defined recurrent acute rhinosinusitis (RARS) as four or more acute bacterial episodes yearly, with symptom-free intervals [96]. The 2020 EPOS guidelines added that RARS diagnosis requires confirmed acute post-viral sinusitis via endoscopy and/or imaging [94]. The ICAR consensus also emphasizes sinus lavage fluid culture [97]. No consensus exists on diagnosis or optimal management [98]. These patients often progress to chronic forms, necessitating surgery; thus, high-volume saline irrigation is a reasonable preventive strategy. It is effective as first-line paediatric therapy, reducing the need for endoscopic sinus surgery [99], particularly in children adhering to the regimen [100].

### 3.6. Chronic Sinusitis

Chronic rhinosinusitis (CRS) is a nasal mucosal inflammation persisting beyond three months. Its prevalence is 4.5–12% in Western countries [101]. It is characterized by headache, rhinorrhoea, nasal obstruction, and hyposmia [102], with significant quality-of-life (QoL) [103] impairment due to sleep disruption [104] and fatigue [105]. CRS is classified as having nasal polyps (CRSwNP) or without (CRSsNP). Polyps arise from mucosal hyperplasia and extracellular oedema, affecting 1–4% of adults and 0.1% of children, but prevalence rises to 6–48% in cystic fibrosis [106]. Polyposis worsens QoL by exacerbating obstruction, rhinorrhoea, purulent discharge, and hyposmia/anosmia; untreated, polyp growth may deform the craniofacial skeleton. First-line treatment combines topical nasal steroids, antibiotics, and systemic steroids, but failure often necessitates functional endoscopic sinus surgery (FESS) [102]. Nasal irrigation (NI) has proven its efficacy: early paediatric studies showed saline’s benefit even without gentamicin, highlighting mechanical clearance of pathogens and inflammatory mediators [107]. Later RCTs and a Cochrane review [108] confirmed NI’s role, leading the 2016 ICAR guidelines to strongly recommend high-volume saline (>200 mL) for both CRSwNP and CRSsNP due to its safety, low cost, and efficacy. NI is also advocated for cystic fibrosis [109] and primary ciliary dyskinesia [110,111,112], where stagnant secretions and antibiotic-resistant infections perpetuate lower airway colonization. Recent advances include steroid-enhanced high-volume saline, improving sinus penetration [112], and hyaluronic acid (HA) additives. CRS patients exhibit reduced periciliary HA [113], and high-molecular-weight HA boosts anti-inflammatory, mucosal repair, and mucociliary effects while inhibiting biofilms [76,114,115]—a key factor in therapy-resistant CRS. HA’s role in polyposis may involve aberrant hyaluronidase activity, altering HA’s size and accumulation [106,116,117]. Functional endoscopic sinus surgery (FESS) is essential for both adults [94] and children [118,119] when medical therapy fails in CRSwNP. A key benefit of FESS is ostial widening, enhancing drug delivery [120] to the inflamed mucosa and improving clinical outcomes [54]. Surgical success depends on avoiding postoperative scarring and restenosis from adhesions/synechiae, which may necessitate revision surgery [121]. While intranasal corticosteroid sprays were traditionally used, their low volume limits sinus penetration (even post-FESS) and increases epistaxis risk [122]. In contrast, low-pressure, high-volume irrigations effectively reach the sinuses [108,123]. A meta-analysis of 14 studies demonstrated that steroid-enhanced saline reduces inflammation, symptoms, and endoscopic scores (polyps, discharge, oedema, scarring) [124]. Saline provides mechanical cleansing, while steroids add anti-inflammatory effects [125]. Similarly, a meta-analysis of 13 studies found hyaluronic acid (HA) supplementation lowers complication risks, notably adhesions (OR 0.52; 95% CI = 0.37–0.72) [126]. Combined saline + steroids + HA irrigation is now a gold-standard postoperative regimen.

### 3.7. Empty Nose Syndrome

This syndrome typically develops following radical surgery, most frequently after inferior turbinate resection [127,128]. The loss of turbinate receptors impairs the nose’s ability to filter, warm, and humidify inhaled air, leading to symptoms of nasal dryness, dyspnoea, and headaches [129]. Turbinates are crucial for nasal homeostasis, and their excessive removal results in empty nose syndrome [130].

Conservative management aims to improve patients’ quality of life and includes nasal hydration gels and optimized rhinosinus care using saline solutions enhanced with xylitol and hyaluronic acid [131].

### 3.8. Allergic Rhinitis (AR)

It is known that saline irrigation significantly alleviates allergic rhinitis (AR) symptoms in children and adults [132,133,134,135], with 78–100% paediatric tolerance [136]. As an adjunct therapy, it reduces antihistamine and steroid use across all age groups. Hypertonic saline (2.7% NaCl) demonstrates superior efficacy versus isotonic solutions. A randomized trial of 220 children (5–9 years) showed hypertonic saline (20 mL twice daily) reduced all AR symptoms (*p* < 0.0001) after 4 weeks, while isotonic saline only improved rhinorrhoea (*p* = 0.0002) and sneezing (*p* = 0.002). Hypertonic solutions also decreased turbinate/adenoid hypertrophy and middle ear exudate (*p* < 0.0001), reducing antihistamine use. Compliance was excellent, with no adverse events [137]. Saline irrigation enhances steroids’ effectiveness by clearing mucus for better drug–mucosa contact [138,139]. In steroid-free AR patients, high-volume irrigation (125–176 mL three times daily) prevents pollen-season IgE elevation [140], suggesting anti-inflammatory effects beyond allergen removal. By eliminating inflammatory mediators [140], NI reinforces mucosal barriers, reduces allergen penetration, and limits IgE production—mirroring corticosteroid mechanisms [39]. Optimal results occur with lukewarm saline (40 °C) [141], which suppresses mast cell degranulation and maintains lower histamine levels for 6 h [139]. Hyaluronic acid additives enhance mucociliary clearance of allergens and inflammatory mediators [142].

### 3.9. Gestational Rhinitis 

Gestational rhinitis (GR) is a pregnancy-specific nasal obstruction developing in the second/third trimester and resolving postpartum. It should not be confused with rhinitis during pregnancy, which encompasses pre-existing conditions (allergic, drug-induced, vasomotor) that persist throughout gestation [143,144,145,146,147,148,149,150,151,152]. Pre-existing rhinitis typically persists unchanged during pregnancy, though 30–45% of patients experience symptoms’ exacerbation or improvement [144]. Treatments are limited because of the teratogenic risks. Specifically, the use of oral steroids in the first trimester can increase cleft palate risk [145]. Oral decongestants can be related to foetal cardiac or limb abnormalities [146]. Preferred options are represented by loratadine/cetirizine (antihistamines) and topical steroids, such as budesonide [147,148]. It is estimated that 60% of women discontinue medications without medical consultation yet readily adopt NI [149,150]. The prevalence of GR is 9–22% [151,152,153]. It is related to snoring, sleep apnoea, pre-eclampsia, gestational hypertension, intrauterine growth restriction, and lower Apgar scores [154,155]. Hormonal-mediated mucosal hypertrophy owes its pathophysiological basis to placental hormones, such as progesterone-induced vasodilation [156,157,158]. Its risk factors are overall smoking [159] and mite allergy [160]. The therapeutic approach is based on lifestyle modifications, particularly exercise, weight control, and 30–45° head elevation during sleep. The use of saline NI represents a fundamental tool: it reduces symptomatic relief [151,161], improves Eustachian tube function [162], reduces pollinosis impact [150], and decreases topical decongestant use (preventing rebound rhinitis) [163]. Moreover, it is safe for both gestational and pre-existing rhinitis [164]. Some studies deepened the use of hypertonic saline NI (3 times daily for 6 weeks). It significantly improved symptoms (*p* < 0.001) and rhinomanometry values (*p* = 0.006) and it is used with excellent compliance and no adverse effects [150].

## 4. Physicians’ Attitude to Nasal Lavage Prescription and Patients’ Adherence to Therapy

Upper respiratory tract diseases are frequent and significantly impact patient quality of life, expenditure on medical resources, and antibiotic use. Nasal saline irrigation represents an adjunctive therapy for upper respiratory tract diseases, and its popularity is growing. A study to evaluate the use of NI as an adjunctive therapy for upper respiratory tract conditions among family physicians in Wisconsin, USA, documented that more than 90% prescribed it for chronic rhinosinusitis and more than half for acute bacterial rhinosinusitis, upper respiratory tract infections, and allergies [165]. Most respondents recommended using NI before antibiotics as a temporary mechanism in bacterial rhinosinusitis. Most physicians recommend NI to adults and children older than seven. The biggest obstacle to the routine recommendation of saline nasal irrigation in children is the assumption by both physicians and parents that children will not tolerate it, especially when they are young. In one survey, only 28% of parents believed their child would tolerate nasal saline irrigation when it was first described to them [62]. A meta-analysis conducted using Medline and Embase databases from January 1946 to June 2015 on the use of lavage in children aged 4 to 12 years with allergic rhinitis identified 40 papers unequivocally documenting that nasal saline irrigations were effective, accepted, and tolerated in most children (78–100%) [136]. In conclusion, saline nasal irrigation is well-tolerated in both adults [108] and children [10], even for treatments of several months [100].

## 5. Risks Related to the Use of NI

A survey carried out using the Delphi method in a group of physiotherapists and physicians experienced in performing nasal irrigation in neonates concluded that only “osteo-meningeal fracture with cerebrospinal fluid leakage” and “ineffective cough or inability to cough” were absolute and relative contraindications, respectively. Swallowing disorders came close to consensus (70% agreement) to be considered an absolute contraindication. Furthermore, experts stated that laryngomalacia or respiratory insufficiency should not be regarded as contraindications to performing nasal irrigation, and just over 60% suggested avoiding this procedure in the case of acute otitis and epistaxis [52]. In adults, contraindications for nasal irrigation with saline include incompletely healed facial trauma, such that saline could potentially leak into other planes or tissue spaces, and conditions associated with an increased risk of aspiration, such as significant intentional tremor or other neurological or musculoskeletal problems [43].

## 6. Conclusions

**Figure 2:**
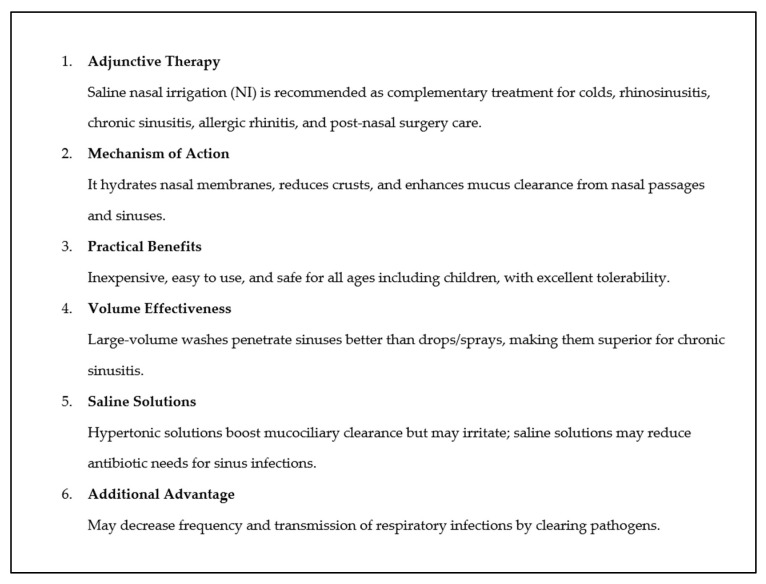
Key points about NI.

The nasal cavity is the primary entry point for pathogens, allergens, and pollutants. Some conditions are known to cause the occurrence of a microbial infection. The physiological changes that occur with NI make this therapeutic modality attractive and additive in many sinonasal pathological conditions for therapeutic and preventive purposes [166] (Figure 2) (Table 1).

NI reduces the adverse effects of these noxae, is easy to perform, and is well accepted by adults, children, and infants. By mechanically decreasing the concentration of these noxious agents at the portal of entry, NI mitigates tissue damage through pathogen/allergen dilution, inflammatory mediator clearance, and mucosal barrier preservation. Contraindications are very rare.

## Figures and Tables

**Table 1:** Key Studies on NI in clinical practice.

	Year	Author	Country	Number of Subjects	Indication for NI	Study Design	Type of NI	Saline	Volume	Duration	Main Results	Adverse Events
Nasal congestion	2016	Schreiber et al. [57]	Italy	133 infants (0–12 years)	Bronchiolitis with respiratory symptoms	Observational study	NI with Isotonic vs. NI with Hypertonic	Isotonic/ hypertonic	1 mL/nostril, once day	Not reported	Improvement in SatO2 after NI with isotonic solution after 15 min (*p* < 0.001)	None
Prevention of respiratory infections	2008	Slapak et al. [11]	Czech Republic	401 children (6–10 years)	Prevention and treatment of common cold	RCT	NI with sea water	Isotonic	Not reported	Not reported	Reduced recurrences, sick days, complications (Recurrent illness: 31% vs. 75%; absences 17% vs. 35%)	None reported
	2004	Tano, L. et al. [65]	Sweden	108 young adults on military service	Prevention of symptoms of common cold in a population of otherwise healthy adults	Clinical trial	Nasal spray with isotonic solution	Isotonic	Not reported	Daily spray for 10 weeks	Significant reduction in days with nasal discharge or stuffy nose (*p* = 0.027)	None
Recurrent infections	2013	Macchi et al. [76]	Italy	75 children	Recurrent respiratory infections	RCT	NI with isotonic solution vs. isotonic solution + hyaluronic acid	Isotonic	9 mg of hyaluronic acid in 3 mL of saline OR 6 mL of saline × 2/day	15 days/month for 3 months	Improved ciliary clearance, adenoid hypertrophy, bacterial clearance, reduced rhinitis duration (*p* < 0.001)	None
	2022	Baxter et al. [84]	USA	79 COVID + patients (>55 years)	Reduce hospitalization	RCT	NI saline solution + sodium bicarbonate vs. saline solution + povidone	Hypertonic	240 mL	Twice a day for 14 days	8-fold lower hospitalization risk; mortality 0% vs. 1.5%	11 reports of irritations
	2022	Gutiérrez-García et al. [87]	Mexico	Healthcare workers	Prevention of COVID-19 infection	RCT	NI called neutral electrolized water (SES)	Hypertonic	4 sprays of 0.4 mL × 3/day or 10 mL by gargling for 60 s × 3/day	4 weeks	Decrease in COVID-19 incidence in the group that adhered to protocol with SES (1.2% vs. 18.8% of control group)	None
Acute sinusitis	2002	Rabago et al. [92]	USA	79 adults with acute sinusitis	Patients with a history of sinusitis	RCT	NI with hypertonic solution	Hypertonic	1 irrigation/day	6 months	Improvement in sinus-related quality of life (*p* ≤ 0.05), decreases symptoms, and decreases medication use in patients with frequent sinusitis	None
	2002	Karadag. [36]	Turkey	Not reported	Acute sinusitis	Clinical trial	Drops of isotonic solution	Isotonic	4 drops/nastril × 4/day	Until remission of symptoms	Reduction in inflammatory mediators, improvement in nasal drainage	None
	2017	Ciofalo, A. et al. [93]	Italy	48 adults with acute sinusitis and treated with Levofloxacin and Prednisone	Acute sinusitis	RCT	NI with isotonic solution vs. NI with isotonic solution + sodium hyaluronate	isotonic	3 mL of saline + sodium hyaluronate or 6 mL of saline, both × 2/day	30 days	Treatment with sodium hyaluronate + saline solution brought about a significant improvement in global assessment of subjective symptoms, normalization of mucociliary transport time and reduction in neutrophil count on nasal cytology	None
Acute recurrent sinusitis	2021	Saltagi, M.Z. et al. [98]	USA	890 patients	Acute recurrent sinusitis	systematic Review	Isotonic solution, sometimes combined with other treatments (antibiotics, intranasal glucocorticoids, decongestants)	Isotonic	Non-standardized	Non-standardized	Positive trend in the combined use of saline irrigation and medical therapy for symptom control, quality of life, and prevention of relapses	None
Chronic rhinosinusitis (CRS)	2011	Wei, J.L. et al. [107]	USA	40 children with CRS	Treatment of chronic rhinosinusitis	RCT	NI with isotonic solution vs. NI with isotonic solution + gentamicin	Isotonic	1 irrigation/day (volume not reported)	6 weeks	Improvement in quality of life, decrease in Lund–Macay score, reduced need for surgery	None
	2020	Thanneru, M. et al. [125]	India	60 patients post-FESS	Control of local inflammation in post-FESS patients with allergic rhinosinusitis with polyps.	RCT	NI with isotonic solution vs. isotonic solution + budesonide	Isotonic	250 mL of saline OR 250 mL of saline + 2 mg of budesonide × 2/day	10 weeks post-FESS	Improvement in quality of life, improvement in endoscopy (Lund–Kennedy score), decrease in severity of symptoms after NI with budesonide (*p* < 0.001)	None
Empty-nose syndrome (ENS)	2011	Modrzyński [128]	Poland	3 patients affected by ENS	Treatment of ENS	Pilot clinical study	Subcutaneous injections of hyaluronic acid gel	/	Not reported	Not reported	Improvement in ENS symptoms	None
Allergic rhinitis	2012	Jeffe, J. et al. [62]	USA	61 children (<18 years)	Allergic rhinitis	Retrospective observational Study	NI with isotonic solution	Isotonic	100 mL × nostril × 2/day	2–4 months	Most children tolerated NI. Improvement in nasal symptoms (*p* < 0.001)	12% mild AE (ear pain, cough, nausea)
	2012	Marchisio et al. [137]	Italy	220 children (5–9 years)	Allergic rhinitis	RCT	NI with isotonic solution vs. NI with hypertonic solution	Isotonic/hypertonic	20 mL/nostril × 2/day	4 weeks	Significant reduction in all symptoms with hypertonic solution (*p* < 0.0001), reduction in rhinorrhoea (*p* = 0.0002) and sneezing (*p* = 0.002) with isotonic solution	None
	2014	Nguyen, S.A [138]	USA	40 adult patients with allergic rhinitis treated with intranasal corticosteroids	Allergic rhinitis already on intranasal corticosteroid pharmacotherapy	Prospective, non-randomized study	NI with isotonic solution	Isotonic	Low pressure, high volume × 2/day	8 weeks	Significant (*p* < 0.001) reduction in mini-Rhinoconjunctivitis Quality of Life Questionnaire	None
Gestational rhinitis	2010	Garavello et al. [150]	Switzerland	45 pregnant women with seasonal allergic rhinitis	Allergic rhinitis in pregnant women	RCT	NI with hypertonic solution	Hypertonic	NI with hypertonic × 3/day	6 weeks	Improvement in rhinitis symptoms (*p* < 0.001), reduction in the use of oral antihistamines (*p* < 0.001), reduction in nasal resistance measured by rhinomanometry (*p* = 0.006)	None

## Data Availability

No new data were created or analyzed in this study.
